# The Role of Vitamins in Non-Alcoholic Fatty Liver Disease: A Systematic Review

**DOI:** 10.7759/cureus.16855

**Published:** 2021-08-03

**Authors:** Rose Anne M Abe, Anum Masroor, Arseni Khorochkov, Jose Prieto, Karan B Singh, Maduka C Nnadozie, Muhammad Abdal, Niki Shrestha, Lubna Mohammed

**Affiliations:** 1 Research, California Institute of Behavioral Neurosciences & Psychology, Fairfield, USA; 2 Psychiatry, California Institute of Behavioral Neurosciences & Psychology, Fairfield, USA; 3 Psychiatry, Psychiatric Care Associates, Englewood, USA; 4 Medicine, Khyber Medical College, Peshawar, PAK; 5 Internal Medicine, California Institute of Behavioral Neurosciences & Psychology, Fairfield, USA; 6 Emergency Medicine, California Institute of Behavioral Neurosciences & Psychology, Fairfield, USA

**Keywords:** vitamins, vitamin a, vitamin b, vitamin c, vitamin d, vitamin e, vitamin k, non-alcoholic fatty liver disease, nafld, vitamins and nafld

## Abstract

Non-Alcoholic Fatty Liver Disease (NAFLD) emerged as the most prevalent liver disorder contributing significantly to disease burden worldwide. It manifests as a broad spectrum of hepatic damage with varying severity ranging from less serious steatosis to a more severe Non-Alcoholic Steatohepatitis (NASH), with or without fibrosis, cirrhosis, and hepatocellular carcinoma. Vitamins, on the other hand, are micronutrients that are vital for healthy well-being. Some studies have linked liver diseases with hypovitaminosis; however, there are still some gaps about the basis of their correlation. Hence, this systematic review aims to discuss the role of vitamins in the pathogenesis of NAFLD and explore their hepatoprotective potential that may benefit clinicians in managing this condition.

This systematic review searched for studies indexed in the PubMed, PubMed Central, Medline, Google Scholar, and ScienceDirect databases. Inclusion and exclusion criteria were applied, duplicates were removed, and meticulous screening of articles was done systematically. Out of 729 unique studies generated using the search strategy, 17 were finally included after thorough review and quality appraisal.

NAFLD is not simply an outcome of insulin resistance and metabolic derangements; instead, it is a disease with complex underlying pathogenesis. Moreover, vitamin deficiency has been associated with NAFLD development and increased susceptibility to more severe liver damage. Derangement in vitamins correlates to the lipotoxic hepatic environment, altered immune system, unwarranted inflammation, oxidative stress, gene mutations, epigenetic modification, and gut dysbiosis seen in NAFLD. As they influence several pathophysiologic processes in the liver, vitamins A, B3, B6, B9, B12, C, D, and E are promising potential options that can impact NAFLD management. However, more well-designed studies conducted in the human population are still necessary to establish their efficacy and safety as therapeutic agents.

## Introduction and background

Liver disorders dramatically contribute to significant disease burden worldwide, with Non-Alcoholic Fatty Liver Disease (NAFLD) emerging as the most prevalent globally and affecting 20-30% of the general population [1]. Specifically, it affects 20-35% of adults [2], 15% of children [3] and reaches as high as 80% of obese individuals [4]. The cases also rise significantly in patients with a background Type 2 Diabetes Mellitus and hyperlipidemia due to its association with insulin resistance and metabolic impairment. Nevertheless, NAFLD can also affect individuals with normal weight and those without metabolic disorders, accounting for about 16% [2]. In addition, it causes an increase in mortality rate and liver transplantations, especially in the United States [5]. Considering that NAFLD is a silent disease with very few manifestations until the later stages, the actual disease burden may be higher than reported [6].

NAFLD is characterized by increased lipid deposition within the hepatocytes in individuals without a history of excessive alcohol intake, that is <30 g/day for men and <20 g/day for women, after excluding other causes of chronic liver diseases [2]. NAFLD manifests as a wide range of hepatic damage with varying severity ranging from simple steatosis to a more severe Non-Alcoholic Steatohepatitis (NASH), with or without fibrosis, cirrhosis, and potentially hepatocellular carcinoma [7]. Although ultrasound can usually diagnose NAFLD, it is only sensitive once the level of fat deposition exceeds 33% [2]. Currently, the gold standard for diagnosis is still liver biopsy [8]. Histologically, NAFLD is described as excessive lipid accumulation in more than 5% of hepatocytes. On the other hand, NASH presents with steatosis in combination with inflammation and ballooning, with or without fibrosis [9]. Due to evidence of hepatic cell death, inflammation, fibrogenesis, and reactive species, patients with NASH have an increased risk for hepatocellular carcinoma and may require liver transplantation [6]. 

Because of the clinical implications of NAFLD, it is crucial to establish its pathogenesis and define the interventions necessary to prevent its serious complications. However, the mechanisms underlying NAFLD and its disease progression are still unclear today. The multiple parallel-hit hypotheses postulate that the disturbance of liver homeostasis in NAFLD is caused by an interplay of lipid and glucose deregulation (i.e., insulin resistance) and mitochondrial and endoplasmic reticulum dysfunction increasing reactive oxygen species production [10]. In addition, contributions from innate immunity, gut microbiota, genetic determinants, epigenetic mechanisms, environmental factors, cytokines, diet, and lifestyle habits are also relevant [10]. Besides the correlation with metabolic syndrome and insulin resistance, NAFLD is also associated with chronic kidney disease [9] and cardiovascular disease [11] complications, thus should be addressed early on.

NAFLD management continues to pose challenges for physicians because there is presently no approved effective pharmacotherapy for this condition. The current standard of care is geared towards lifestyle improvement involving weight loss, increased physical activity, and a relatively low-calorie diet (with caloric quantity proportional to energy consumption) [8]. Although these core therapeutic interventions are helpful, strict compliance and long-term effort have been an issue for many patients [1]. Moreover, some studies have highlighted that reducing high-energy food and engaging in a more active lifestyle are inadequate in preventing and treating NAFLD [5]. This proves that NAFLD is not simply a consequence of metabolic syndrome and insulin resistance [10]. Thus, further intensive research is still necessary to establish its pathogenesis, diagnosis, and treatment. 

Some studies attempt to explore how specific macronutrients and micronutrients, including vitamins, contribute to the development and possible alleviation and treatment of NAFLD [4,5,8,9]. Moreover, identifying novel potential targets that can serve as indirect therapies for NAFLD is still a research subject. Vitamins, whether lipid-soluble (A, D, E, K) or water-soluble (group B and C), are essential micronutrients for the maintenance of health [5]. While some studies have found an association between chronic liver diseases and hypovitaminosis [3], insufficient data describes the mechanisms behind their correlation. Hence, this study aims to provide a broader discussion of the crucial role of vitamins in the pathogenesis of NAFLD and explore their hepatoprotective potential in managing this condition.

## Review

Methods

Protocol

This systematic review was conducted following the Preferred Reporting Items for Systematic Review and Meta-analysis (PRISMA) guidelines.

Inclusion and Exclusion Criteria

Articles included in this review were published between 2016 and 2021, all written in English and readily available online. These studies were conducted on human and animal subjects. All types of clinical studies were included.

Search Strategy

This integrative review searched for articles indexed in the PubMed, PubMed Central, Medline, Google Scholar, and ScienceDirect databases up to June 25, 2021, utilizing Medical Subject Headings (MeSH) terms and regular search keywords such as "Vitamins," "Vitamin A," "Vitamin B," "Vitamin C," "Vitamin D," "Vitamin E," "Vitamin K," and "Non-alcoholic Fatty Liver Disease," which were used both individually and in combination. Articles generated were further screened to determine their relevance to the focus of this study. Table 1 and Table 2 demonstrate the search strategy using regular keywords and MeSH terms, respectively.

**Table 1:** Database Search Results Using Regular Keywords

Regular Keywords	Total Articles	Total Articles after application of Inclusion/Exclusion Criteria
Vitamins and Non-alcoholic Fatty Liver Disease	3863	688
Vitamin A and Non-alcoholic Fatty Liver Disease	3060	413
Vitamin B and Non-alcoholic Fatty Liver Disease	2777	419
Vitamin C and Non-alcoholic Fatty Liver Disease	2857	408
Vitamin D and Non-alcoholic Fatty Liver Disease	2664	504
Vitamin E and Non-alcoholic Fatty Liver Disease	2718	494
Vitamin K and Non-alcoholic Fatty Liver Disease	1465	393

**Table 2:** Database Search Results Using MeSH Terms MeSH - Medical Subject Headings

MeSH terms	Total Article	Total Articles after application of Inclusion/Exclusion Criteria
("Vitamins"[MeSH]) AND "Non-alcoholic Fatty Liver Disease"[MeSH]	56	14
("Vitamin A"[MeSH]) AND "Non-alcoholic Fatty Liver Disease"[MeSH]	31	9
("Riboflavin"[MeSH]) AND "Non-alcoholic Fatty Liver Disease"[MeSH]	2	1
("Niacin"[MeSH]) AND "Non-alcoholic Fatty Liver Disease"[MeSH]	8	1
("Adenine"[MeSH]) AND "Non-alcoholic Fatty Liver Disease"[MeSH]	6	2
("Pantothenic Acid"[MeSH]) AND "Non-alcoholic Fatty Liver Disease"[MeSH]	1	1
("Vitamin B 6"[MeSH]) AND "Non-alcoholic Fatty Liver Disease"[MeSH]	5	2
("Folic Acid"[MeSH]) AND "Non-alcoholic Fatty Liver Disease"[MeSH]	21	7
("Vitamin B 12"[MeSH]) AND "Non-alcoholic Fatty Liver Disease"[MeSH]	8	1
("Ascorbic Acid"[MeSH]) AND "Non-alcoholic Fatty Liver Disease"[MeSH]	23	7
("Vitamin D"[MeSH]) AND "Non-alcoholic Fatty Liver Disease"[MeSH]	148	51
("Vitamin E"[MeSH]) AND "Non-alcoholic Fatty Liver Disease"[MeSH]	168	46
("Vitamin K"[MeSH]) AND "Non-alcoholic Fatty Liver Disease"[MeSH]	3	0

Data Extraction and Bias Evaluation

Titles, abstracts, and full texts of relevant studies were scrutinized for eligibility. Extracted from each article include the year of publication, the purpose of the study, and findings that mainly focus on vitamins and their role in pathogenesis and potential management of NAFLD. Moreover, quality appraisal of included studies was done using Cochrane Risk Assessment tools such as Newcastle-Ottawa tool for Non-RCT (randomized controlled trials) and Observational Studies, PRISMA checklist for systematic reviews, Scale for the Assessment of Narrative Review Articles (SANRA) checklist for traditional reviews, and Systematic Review Centre for Laboratory animal Experimentation (SYRCLE) Assessment tool for animal studies. This was meticulously performed independently by at least two authors. After careful analysis and quality check, only moderate to high-quality studies were included in the final review. 

Results

Search Outcome

**Figure 1:**
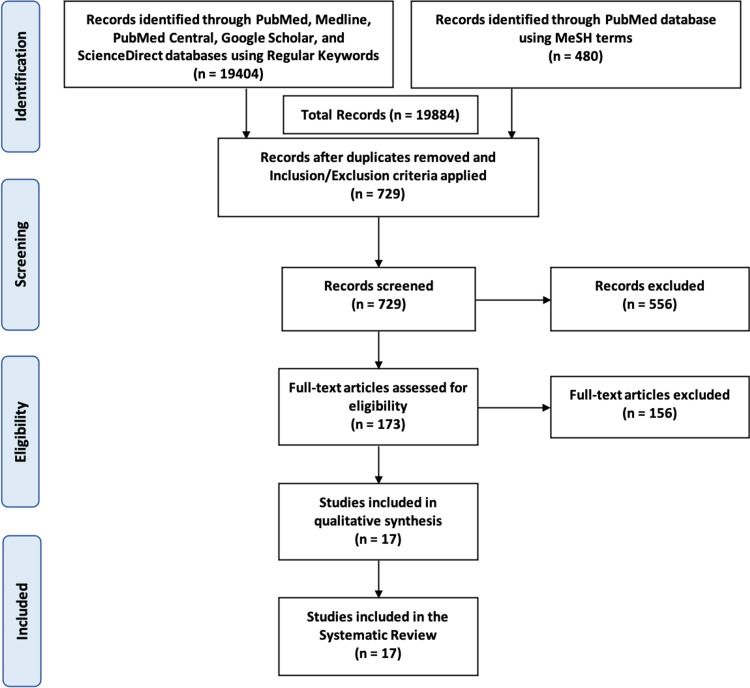
PRISMA Flow Diagram PRISMA - Preferred Reporting Items for Systematic Review and Meta-analysis, MeSH - Medical Subject Headings

After using regular search keywords and MeSH terms, there were 19884 articles generated from PubMed, PubMed Central, Medline, Google Scholar, and ScienceDirect databases. These studies were filtered based on inclusion and exclusion criteria, and duplicates were removed. The remaining 729 studies were further screened manually through the titles and abstracts to determine their relevance to the focus of this study, thereby excluding 556 articles. Afterward, the full texts of 173 articles were then assessed for eligibility. After a thorough review and quality appraisal, 17 articles were finally included in this review. Figure 1 shows the PRISMA Diagram to demonstrate the search process.

Discussion 

This section discusses the pathophysiology of NAFLD and summarizes studies exploring the role of vitamins in the development and progression of this condition. Moreover, this discussion provides insights into the impact of vitamins as potential targets for NAFLD management. 

Pathogenesis of NAFLD

**Figure 2:**
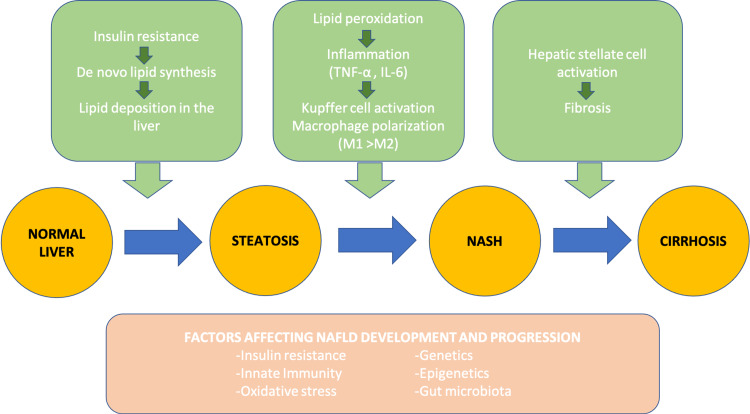
Pathophysiologic Processes in NAFLD Development and Progression Adapted From Source: Chen et al. [9] and Nagashimada et al. [10] TNF-α - tumor necrosis factor-alpha, IL-6 - interleukin-6, M1 - classically activated macrophages, M2 - alternatively activated macrophages, NASH - Non-Alcoholic Steatohepatitis, NAFLD - Non-Alcoholic Fatty Liver Disease

The concept of NAFLD has been initially described via the two-hit hypothesis, which states that obesity or diabetes-induced steatosis and increased hepatic uptake of free fatty acids (the first hit) make the liver more susceptible to further damage due to oxidative stress, lipid peroxidation, and release of pro-inflammatory cytokines (the second hit) [5]. However, recent studies have shown that NAFLD is not simply a result of insulin resistance and metabolic syndrome; instead, it is a multifactorial disease. In line with this, multiple parallel hit hypothesis states that the combination of diverse factors such as insulin resistance, adipokine secretion, oxidative stress, lipid peroxidation, mitochondrial damage, endoplasmic reticulum stress, intestinal microbiota, innate immunity, genetics, and epigenetic mechanisms ultimately cause liver injury leading to the progression of NAFLD [2,9,10]. Figure 2 shows the factors contributing to NAFLD development and severity.

Insulin Resistance and NAFLD

Insulin resistance is a fundamental factor in NAFLD pathogenesis [1]. Due to the impairment of the anti-lipolytic action of insulin, excessive free fatty acid (FFA) is produced, resulting in overwhelming FFA delivery to the liver and de novo lipogenesis, prompting insulin resistance [10]. Factors that specifically contribute to fat metabolism imbalance are dysregulation of insulin signaling pathways such as sterol regulatory element-binding protein 1, fatty acid translocase cluster differentiation protein 36 (FAT/ CD36), and hormone-sensitive lipase, which leads to triglyceride imbalance, fatty acid mitochondrial oxidation, and lipoprotein excretion and transport [12]. Moreover, the excessive exposure to fatty acids leads to adipocyte exhaustion and further liver damage by suppressing adiponectin and stimulating the release of other inflammatory and pro-fibrotic cytokines such as leptin, resistin, tumor necrosis factor-alpha (TNF-α), and interleukin-6 (IL-6) [12,13]. Adiponectin is an adipose-specific secretory adipokine that has anti-inflammatory and anti-diabetic properties. In addition to antagonizing inflammation by inhibiting nuclear factor-kappa B (NFκB) action and TNF-α expression [2], it enhances oxidation and lipid transfer of FFAs to inhibit unwarranted binding of FFAs to their respective receptors in the hepatocyte and subsequent fat accumulation [10]. On the contrary, pro-inflammatory adipokines, TNF-α and IL-6, inhibit adiponectin but upregulate leptin levels leading to anabolic pathway inhibition [13]. Leptin, moreover, activates hepatic stellate cells (HSC), amplifying inflammation and fibrogenesis in the liver [2,12].

Innate Immunity and NAFLD

The liver contains a collection of immune cells from the monocyte and macrophage lineage. Dendritic cells, Kupffer cells, Natural killer cells, and hepatic stellate cells are components of innate immunity that have influenced NAFLD pathogenesis [5]. Kupffer cells and recruited macrophages secrete inflammatory cytokines such as TNF-α, interleukin-1 beta (IL-1β), and IL-6, prompting systemic insulin resistance and eventually NASH [10]. Macrophages are divided into classically activated macrophages (M1) and alternatively activated macrophages (M2) [9]. Previous in vitro and in vivo studies have demonstrated that the pathology of NAFLD is associated with dysregulation and polarization of M1/M2-like macrophages wherein M1-like macrophages initiate and sustain inflammation, and M2-like macrophages attenuate chronic inflammation [10]. This phenomenon is also associated with insulin resistance and metabolic disorders such as obesity and diabetes [9,10]. The mechanisms leading to increased infiltration of macrophages into visceral adipose tissue are not entirely clear. However, it is known that the binding of chemokines such as monocyte chemoattractant protein 1 (MCP-1), also known as C-C motif ligand (CCL) 2, with its receptor induces recruitment of macrophages in adipocyte and hepatocyte, leading to liver steatosis and insulin resistance in obese patients [2,10]. 

Oxidative Stress and NAFLD

Oxidative stress is defined as the imbalance between the reactive oxygen species (ROS) production and the scavenging capacity of the antioxidant system (including superoxide dismutase and catalase) in favor of the former [10,14]. At relatively low levels of antioxidant repair enzymes, hydrogen peroxide generated by Fenton reaction and induced by elevated iron levels in NASH can enhance fatty acid oxidation and cause deleterious effects to the electron transport chain (ETC) and the mitochondrial deoxyribonucleic acid (DNA), leading to mutations and cellular apoptosis [13]. Furthermore, mitochondrial proliferation and differentiation, primarily regulated by peroxisome proliferator-activated receptor-gamma-coactivator-1 alpha (PGC-1α), can be impaired in NASH [12]. Reportedly, patients with steatosis and metabolic disorders have decreased antioxidant defenses and increased lipid peroxidation owing to higher levels of lipid peroxides (thiobarbituric acid-reactive substances [TBARS]) compared to healthy controls [10]. This is a consequence of FFA overload that overwhelms mitochondrial energy reserves, leading to fatty acid accumulation and metabolism by peroxisomes and microsomes [12,13]. 

Moreover, hyperinsulinemia inhibits mitochondrial oxidation of fatty acids. Insulin resistance upsurges peroxisomal oxidation since insulin is the principal inhibitor of cytochrome P450 4A (CYP4A), a significant enzyme in this pathway [13]. Amplified cytotoxic ROS production may deplete antioxidant molecules, such as glutathione, and influence the release of pro-inflammatory and fibrogenic cytokines, such as TNF-α, transforming growth factor-beta (TGF-β), Fas ligand, and interleukin-8 (IL-8) [14]. Enhanced lipid peroxidation also leads to the formation of aldehyde byproducts, such as malondialdehyde (MDA), which has a longer half-life than ROS and leads to further oxidative stress [13]. 

Genetics and NAFLD

Some studies supported the impact of genetics on hepatic steatosis and inflammatory changes or fibrosis. Genome-wide studies have identified some association between NAFLD susceptibility and Transmembrane 6 superfamily member 2 (*TM6SF2*) and Patatin-like phospholipase domain-containing 3 (*PNPLA3*) [5,15]. Together with visceral obesity, insulin resistance, high cholesterol, and fructose intake, these genes are also the most prevalent risk factors for lean NAFLD, representing a subpopulation of patients with fatty liver but normal body mass index (BMI) [16]. *PNPLA3*, moreover, is a gene that encodes for triacylglycerol lipase that mediates lipid hydrolysis and maintains lipid homeostasis by maintaining a balance between energy consumption and storage in the fat tissue [13]. A single nucleotide polymorphism (SNP) of this gene can lead to steatosis, inflammation, fibrosis, and even hepatocellular carcinoma (HCC) [15]. On the other hand, *TM6SF2* activity is essential for normal very low-density lipoprotein (VLDL) secretion; however, hepatic expression of this gene is significantly low in NAFLD patients [15]. Another notable gene is G-protein-coupled-receptor 120 (*GPR120*), a receptor for polyunsaturated fatty acids (PUFAs) expressed by adipocytes, Kupffer cells, and hepatocytes. *GPR120* 270H carriers have abnormal liver function tests (LFTs) due to hepatocyte injury [12]. Lastly, peroxisome proliferator-activated receptor-gamma (PPARγ), a molecular target of diabetic drug glitazones, is significantly expressed in adipose tissues and regulates adipocyte differentiation and hepatic fatty acid influx and efflux [12]. Mutations in these genes ultimately lead to hepatic steatosis. 

Epigenetics and NAFLD

Recently, some studies have featured the role of epigenetic mechanisms in NAFLD. For example, research done by Walle et al. shows that genetic variants of fatty acid desaturase (FADS)2, which is significantly expressed in the liver, contribute to the pathogenesis of NAFLD by modifying fatty acid metabolism through DNA methylation [17]. In addition, changes in epigenetic mechanisms explain the immediate effect of maternal obesity seen in the offspring [5].

Gut Microbiota and NAFLD

Interestingly, recent studies have demonstrated the "gut-liver axis," which links intestinal microbiota and NAFLD. It is primarily due to increased exposure and susceptibility of the liver to gut microbiome changes as it receives 70% of its blood supply from the gut through the portal vein [16]. In addition, it is believed that this association is owed to increased generation of bacteria-derived endotoxin [5] or downregulation of tight junction proteins (zonula occludens-1 and occludin), leading to disruption of gut barrier and translocation of toxins to extra-intestinal tissues [13]. Notably, intestinal permeability doubles in NAFLD and the prevalence of Small Intestinal Bacterial Overgrowth (SIBO) is tripled in these patients [14]. Due to Gram-negative bacteria overgrowth in the intestine, NAFLD patients exhibited a 38-40% rise in their lipopolysaccharide (LPS) serum level compared to other patients without NAFLD [14]. The increased LPS level may also activate an intracellular inflammatory cascade inducing the release of pro-inflammatory cytokines in the Kupffer cells resulting in hepatocellular injury [15]. The intestinal microbiota also releases Toll-like receptor (TLR) ligands such as TLR2, TLR4, and TLR9. These are pathogen-associated molecular patterns (PAMPs) involved in developing NASH in humans and promoting pro-fibrotic pathways in the liver [13]. The gut microbiota also decreases the expression of fasting-induced adipose factor (Fiaf), an inhibitor of lipoprotein lipase, resulting in increased fatty acid uptake and triglyceride accumulation [13,15]. 

Indeed, the underlying pathogenesis of NAFLD is a multitude of complex mechanisms that need further studies for clarification. Since vitamins significantly impact the immune system and have modulatory properties through various means, their role in NAFLD is worth exploring. 

Vitamin A

The role of Vitamin A, also known as retinoic acid, in NAFLD has not been extensively explored. However, studies have shown that patients with NAFLD have Vitamin A deficiency compared to non-diabetic and diabetic non-NAFLD patients [4,5,18-20]. Recently, studies have found that *PNPLA3*, a gene closely associated with NAFLD, harbors retinyl ester hydrolase activity [20]. Mutation of this gene may lead to low serum retinol but increased retinyl esters in the liver of NAFLD patients [1,20]. Moreover, Coelho et al. have demonstrated a significant reduction in serum retinol in the advanced fibrosis stage [4]. The liver, particularly the quiescent hepatic stellate cell (qHSC), is the primary storage site of Vitamin A. As qHSC becomes activated for fibrogenesis, retinoic reserves are lost in the process [4,5,20]. Vitamin A has also been recognized for having antioxidant properties; thus, inadequate intake may lead to the progression of liver damage as oxidative stress contributes to NAFLD pathology [4]. Furthermore, the negative correlation of serum retinoic acid level and markers of liver injury and adiposity (intrahepatic triglyceride and transaminase levels) support the idea that Vitamin A has a role in modifying glucose and lipid metabolism in the liver [5,18]. This is attributed to the ability of retinoic acid to increase the expression of genes promoting fatty oxidation in the liver, such as proliferator-activated receptor-alpha (*PPARα*), fibroblast growth factor 21 (*FGF21*), carnitine palmitoyltransferase I (*CPT1*), and uncoupling protein 2 (*UCP2*) [21]. Although some studies have shown decreased risk of NAFLD in individuals with higher Vitamin A intake [4], precaution must be taken in young children because this supplement may lead to higher adiposity in this population due to stage-dependent effects during development [5].

Vitamin B

There are eight types of compounds belonging to the Vitamin B group; however, only a few have been studied in light of NAFLD conditions. Vitamin B3 (niacin) is significant in lipid metabolism as it acts as a precursor for coenzyme nicotinamide adenine dinucleotide (NAD) and nicotinamide adenine dinucleotide phosphate (NADPH) [21]. In rats fed with an obesogenic diet, niacin supplementation causes increased redox potential, reduction in hepatic and serum triglyceride content, and amelioration of hepatic steatosis [5]. In another study involving a diet-induced mouse model of liver fibrosis, supplementation of nicotinamide riboside (NR), a NAD precursor, attenuated hepatic stellate cell activation resulting in decreased liver fibrosis [22]. Moreover, in vitro study involving palmitate-incubated Hep G2 cells, primary human liver cells, and niacin showed inhibition of lipid deposition, decreased NADPH oxidase activity, low IL-8 cytokine level, and reduced ROS production [18]. Conversely, other interventional studies have shown that long-term niacin supplementation can lead to insulin resistance; thus, it may exacerbate NAFLD's already reduced insulin sensitivity [5].

Dysregulation in Vitamin B9 (folate or folic acid) metabolism has been implicated in NAFLD-related comorbidities such as obesity, Type 2 Diabetes Mellitus, and metabolic syndrome [21]. Furthermore, genetic mutations in the folate pathway are correlated to hyperhomocysteinemia, which promotes lipid accumulation in the liver [21]. A study also reveals that a lower folate level is associated with increased NASH histological severity [3]. Moreover, another case-control study shows that folic acid deficiency is significantly higher in patients with a fatty liver; however, it is not confirmed if this vitamin deficiency contributes to the incidence, exacerbation, and persistence of fatty liver disease [23]. This is likely due to the small sample size involved. Also, performing a confirmatory liver biopsy is not feasible in their study due to ethical barriers. Nevertheless, the hepatoprotective benefit of folic acid has been linked to its ability to restore activation of adenosine monophosphate-activated protein kinase (AMPK), an enzyme that is associated with liver steatosis, insulin resistance, and hyperglycemia when inactivated [21]. Thus, this evidence supports the therapeutic potential of folic acid supplementation in NAFLD patients.

Vitamin B12 (cyanocobalamin) influences DNA synthesis and repair [5] as well as mitochondrial metabolism [18], whose damage is commonly implicated in NAFLD pathogenesis. Vitamin B12 comes into play in this scenario since it is a cofactor for methyl malonyl coenzyme A (CoA) mutase that regulates the transfer of long-chain fatty acyl CoA into the mitochondria [5]. Moreover, the liver is the primary storage site of Vitamin B12. Mahamid et al. have discussed that Vitamin B12 deficiency can be an independent predictor of the severity of NASH histology in terms of disease activity and fibrosis grade [3]. This supports the outcomes of earlier studies stating that there are lower serum Vitamin B12 levels in NAFLD patients than controls, and it correlates with a higher grade of steatohepatitis [3]. Also, it has been shown that there is a higher rate of hyperlipidemia and Type 2 Diabetes Mellitus in offspring of mothers with low Vitamin B12 due to altered PPARα and PPARγ in the liver; however, supplementation of Vitamin B12 can normalize this alteration in the offspring [5,21]. 

Conversely, serum levels of Vitamin B12 in NAFLD subjects are either unchanged or moderately reduced in some literature [18]. In addition, a study done in human subjects has shown no difference in Vitamin B12 levels in NAFLD patients and control subjects [5]. However, this might be due to the small sample size involved. 

Information regarding the association of other Vitamin B compounds and NAFLD is scarce in the literature. However, one study links NAFLD with pyridoxal 5'-phosphate (PLP), the biologically active form of Vitamin B6, which serves as a cofactor for homocysteine catabolism [24]. In this study, Vitamin B6 deficiency prompts accumulation of homocysteine which further leads to stress in the endoplasmic reticulum and activation of transcription factor sterol response element-binding protein 1c and de novo lipid production; thus, they recommend Vitamin B6 to reduce hepatic fat accumulation [24]. Currently, evidence for interplay between Vitamin B and NAFLD is still controversial and has yet to be resolved.

Vitamin C

Owing to its antioxidant properties, Vitamin C (ascorbic acid) plays a role in scavenging free radicals [19]. A cross-sectional study noted an increased incidence of NAFLD in older adults with low dietary intake of Vitamin C [19]. Moreover, low Vitamin C is inversely associated with NAFLD severity [14]. Currently, there are insufficient studies that discuss the mechanisms by which Vitamin C deficiency leads to liver injury. Some reports state that Vitamin C decreases mitochondrial ROS generation, increases the levels of antioxidant enzymes such as superoxide dismutase and glutathione peroxidase, and improves the electron transport chain activity in the liver [16,21]. Also, Vitamin C affects lipid and glucose homeostasis and suppresses visceral obesity and NAFLD by activating PPARα [25]. In addition, a low level of Vitamin C can lead to decreased cholesterol excretion since it serves as a cofactor in the rate-limiting step in bile acid formation [26]. Moreover, ascorbic acid alleviates inflammatory conditions by reducing C-reactive protein, IL-6, and myeloperoxidase [25,26]. Also noted is its potential impact on adiponectin, leading to decreased steatosis and insulin resistance [26]. All of these lead to attempts to explore the therapeutic benefits of ascorbic acid in NAFLD.

In a study conducted on high-fat-diet-induced mice, prophylactic use of low (15 mg/kg per day) and medium (30 mg/kg per day) doses of Vitamin C reduced the risk of NAFLD development, as evidenced by the significantly decreased weight of the body, adipose tissue mass, and steatosis [25]. Another study found significant improvement in the liver fibrosis score of NASH patients after Vitamin C supplementation [4]. Also, the efficacy of Vitamin C in combination with Vitamin E in NAFLD patients has been evaluated in some studies [5,19,26]; however, results are inconclusive, because both are considered antioxidants, it is unclear whether the beneficial contribution is due to individual or combined effects.

Vitamin D

Vitamin D insufficiency has been associated with biopsy-proven NAFLD [5] and liver fibrosis [27]. One study done in morbidly obese patients showed that Vitamin D deficiency is associated with a higher risk of steatosis represented by Fatty Liver Index (FLI) score [7]. Low levels of Vitamin D activate Toll-like receptors, leading to severe liver inflammation and oxidative stress. [9,18]. In chronic hepatic diseases like NAFLD, Vitamin D receptor (VDR) expression is inversely associated with the severity of lobular inflammatory damage [2,7,28].

On the contrary, a recent meta-analysis of six studies showed that a low 25-hydroxyvitamin D [25(OH)D] level is not associated with a higher degree of liver scarring in NAFLD [29]. Since Vitamin D's anti-fibrotic effect depends on VDR genotypes and levels, polymorphisms in VDRs can also explain the inconsistent association of NAFLD with Vitamin D levels [18]. Activation of VDR in liver macrophages and hepatic stellate cells leads to attenuation of hepatic inflammation and fibrosis; conversely, VDR activation in hepatocytes could accelerate lipid accumulation [30]. While some argue that the association between hypovitaminosis D and NAFLD is only due to their high prevalence universally, epidemiological evidence shows that Vitamin D deficiency is more frequently found in NAFLD patients than in the general population [9]. This indicates that hypovitaminosis D and NAFLD share several risk factors; hence they coexist [21].

Vitamin D and Vitamin D receptors participate in the liver, adipose, and gut homeostasis, owing to its notable insulin-sensitizing, anti-inflammatory, and anti-fibrotic effects [11]. For instance, VDR in pancreatic beta cells regulates the insulin gene [11]. In addition, Vitamin D favors glucose uptake in the muscle by intensifying the intracellular expression of the insulin receptor substrate (IRS)-1 and enhancing the insulin-dependent glucose transporter 4 (GLUT-4) on fat tissues [11]. Moreover, besides favoring insulin release from the pancreas, Vitamin D also induces adiponectin release from fat tissue [7]. In a study involving human patients, serum 25-hydroxyvitamin D concentration correlated with low leptin and high adiponectin levels, irrespective of BMI [2]. 

Several in vitro studies on both mouse and human adipocytes demonstrated the anti-inflammatory effect of Vitamin D in decreasing chemokines and cytokines expression via the involvement of p38 Mitogen-Activated Protein (MAP) kinase and the NF-κB classical inflammatory pathway [2]. In addition, Vitamin D exerts anti-fibrotic activity in the liver by inhibiting hepatic stellate cell activation and reducing the expression of fibrogenic factors such as platelet-derived growth factor (PDGF), TGF-β, collagen, alpha-smooth muscle actin (α-SMA), and tissue inhibitors of metalloproteinase-1 [11,18,27]. Moreover, it inhibits monocyte activation and TNF-α and interleukin-1 (IL-1) expression [9]. 

Some research showed that VDR regulates the expression of the tight junctions zona occludens (ZO) proteins 1 and 2 (ZO-1 and ZO-2) through increasing claudin 2 and 12 and decreasing cadherin-17, thus maintaining the adhesion of intestinal epithelial cells [11]. Furthermore, Vitamin D supports gut integrity by repairing tight junctions injured by bacterial lipopolysaccharide and preventing cell death during the inflammatory process [11]. 

Despite the association and benefits found in previous studies, some literature has failed to find a favorable response to Vitamin D supplementation in liver function or histology in NAFLD patients. For instance, a systematic review has seen significant improvement in lipid profile and inflammatory mediators, but not in liver enzymes, anthropometric measures, and glycemic index in NAFLD patients [31]. Furthermore, Vitamin D supplementation is clinically limited because it can lead to hypercalcemia, a risk factor for NAFLD [19]. These controversies can be due to the small population size, different outcome measures, and varying follow-up periods; thus, a more well-designed study with standardized criteria and a larger sample size is warranted. 

Vitamin E

Vitamin E, a lipophilic compound, exists naturally as tocopherol (alpha, beta, gamma, delta) and tocotrienol (alpha, beta, gamma, delta) [6]. Among these, alpha-tocopherol is the most abundant and most potent antioxidant, which acts as scavengers of free radicals [21]. Vitamin E can increase antioxidant enzymes such as superoxide dismutase, catalase, and glutathione peroxidase; conversely, it decreases pro-oxidant contributors such as cellular myelocytomatosis (c-myc) and transforming growth factor-alpha (TGF-α), nitric oxide synthase, and NADPH [19]. It also has an antisteatotic effect owing to its ability to downregulate the hepatic cluster of differentiation 36 (CD36) protein, thus reducing hepatocyte fatty acid uptake and decreasing the pool of lipids for peroxidation [5,18,21]. Moreover, Vitamin E lowers hepatic inflammation and fibrosis by decreasing the expression of pro-apoptotic BCL2 associated X (*BAX*), *TGF-β*, cyclooxygenase-2 (*COX-2*), and matrix metalloproteinase-2 (*MMP-2*) genes [32]. 

Pioglitazone Versus Vitamin E Versus Placebo for the Treatment of Non-Diabetic Patients with Nonalcoholic Steatohepatitis (PIVENS) trial shows that Vitamin E, compared to other interventions, leads to reduction of steatosis and inflammation and improvement in liver histology but not fibrosis [10,21]. In another trial called Treatment of NAFLD in children (TONIC), both metformin and Vitamin E improve the hepatocellular ballooning and NAFLD activity score in children and adolescents with NASH. However, there is no change in liver function test, steatosis, inflammation, or fibrosis [10]. 

In a systematic review and meta-analysis done by Abdel-Maboud et al., Vitamin E supplementation significantly improved alanine transaminase (ALT), aspartate transaminase (AST), fibrosis, and NAFLD activity score (NAS) at early and late follow-up in adult patients and biochemical parameters in the long term follow up in pediatrics [1]. Some studies also evaluated its use in combination with other therapeutic options [33]. However, regression analysis showed that combined interventions did not significantly modify these parameters; hence, Vitamin E is effective on its own and not merely an adjuvant [1]. 

The American Association for the Study of Liver Diseases (AASLD) and the National Institute for Health and Care Excellence (NICE), United Kingdom, recommend the use of Vitamin E in NAFLD treatment [32]. Notably, a dose of 800 IU daily can improve histologic findings in non-diabetic patients with biopsy-proven NASH [5,21]. Although the safety profile of Vitamin E is still in question due to its controversial adverse effects with long-term use such as prostate cancer in men [5] and hemorrhagic stroke [1], the intake of alpha-tocopherol is currently the only intervention that leads to mortality rate drop and transplant-free survival improvement among NASH patients [6].

Vitamin K

Little is known about the role of Vitamin K in lipid metabolism. Although one study described a positive relationship between adult obesity and Vitamin K concentration in fat tissues [5], literature exploring the association of Vitamin K with NAFLD is currently lacking.

Vitamins, thus, have a significant impact on NAFLD pathogenesis and management, as evidenced by various literature. Table 3 summarizes the findings from 17 relevant studies included in this review. 

**Table 3:** Summary of Included Articles NAFLD - Non-Alcoholic Fatty Liver Disease, NASH - Non-Alcoholic Steatohepatitis, ROS - reactive oxygen species, TNF-α - tumor necrosis factor-alpha, TGF-β - transforming growth factor-beta, FLI - Fatty Liver Index, BARD - body mass index, aspartate transaminase/alanine transaminase ratio, and Diabetes Mellitus

Authors	Year	Study Type	Purpose of Study	Results/Conclusion
Chen et al. [9]	2016	Traditional Review	To present molecular mechanisms involved in the pathogenesis of NAFLD and introduce some dietary antioxidants that may be helpful in NAFLD prevention and therapy	Overproduction of ROS and changes in adiponectin, chemokines, TNF-α, and TGF-β are the leading promoters of NAFLD development. Exercise and healthy dietary supplements, including micronutrients, are promising methods to manage NAFLD.
Li et al. [5]	2016	Traditional Review	To discuss the role of Vitamins in NAFLD development and management	Vitamins A, B3, B12, D, and E can serve as targets for NAFLD therapy, although some are linked to adverse effects.
Cimini et al. [2]	2017	Traditional Review	To provide an overview of recent advances in the pathogenesis of NAFLD concerning adipose tissue dysfunction and the pathophysiology linking Vitamin D deficiency with NAFLD and adiposity, and to give a summary of the evidence available on the utilization of Vitamin D supplementation in NAFLD cases	Vitamin D status and obesity have an inverse relationship. Hypovitaminosis D is associated with an unfavorable metabolic and inflammatory profile. Vitamin D's anti-inflammatory and immunomodulatory properties link hypovitaminosis D with the progression of NAFLD.
Saeed et al. [20]	2017	Traditional Review	To discuss the Vitamin A metabolism in NAFLD and its role in the progression of liver disease and therapeutic potential of Vitamin A metabolites	Vitamin A metabolites regulate hepatic glucose and lipid metabolism. It remains unclear whether Vitamin A deficiency contributes to hepatic steatosis, and human data are currently lacking.
Cicero et al. [8]	2018	Traditional Review	To evaluate the effect of nutraceuticals on NAFLD and NAFLD-related parameters	Vitamin E and Vitamin D have positive impacts on NAFLD and NAFLD-related parameters.
Mahamid et al. [3]	2018	Cross-sectional Study	To investigate the correlation between folate and B12 serum levels with NASH severity, based on the fibrosis grade and activity	There is a statistically significant correlation between folate and Vitamin B12 deficiencies with the severity of NASH histology.
Perumpail et al. [19]	2018	Traditional Review	To discuss the role played by Vitamin E in NASH patients	Vitamin E has antioxidant, anti-inflammatory, and anti-apoptotic properties. Also, it is easy to use and well-tolerated. Vitamin E is a logical therapeutic choice in non-diabetic patients with histologic evidence of NASH.
Pickett-Blakely et al. [18]	2018	Traditional Review	To analyze the mechanisms by which micronutrients contribute to NAFLD pathogenesis and determine their role as therapeutic targets for NAFLD	Deficiency or excess of micronutrients deregulates homeostatic and oxidative pathways. Vitamin A and D deficiencies suggest advanced liver disease in cirrhotic patients. Circulating levels of fat-soluble vitamins predict the efficacy of novel NAFLD therapies that target bile acid signaling.
Hariri et al. [31]	2019	Systematic Review	To evaluate the effectiveness of Vitamin D in the treatment of NAFLD	Vitamin D supplements can improve NAFLD through inflammation reduction.
Nagashimada et al. [10]	2019	Traditional Review	To review the pathogenesis of NAFLD on a molecular level and the potential utility of Vitamin E in its prevention and treatment	As an antioxidant, Vitamin E inhibits ROS production, which is implicated in the development of steatohepatitis. Vitamin E may promote liver homeostasis by regulating macrophage polarization, thereby halting the progression of NASH.
Abdel-Maboud et al. [1]	2020	Systematic review, Meta-analysis, and Meta-regression	To examine the efficiency of Vitamin E when used alone or in combination with other interventions for the management of NAFLD	Whether alone or combined, there is a significant improvement in biochemical and histological outcomes using Vitamin E in adults and pediatric patients with NAFLD.
Barchetta et al. [11]	2020	Traditional Review	To discuss pathophysiologic pathways connecting Vitamin D to NAFLD, emphasizing the effects of Vitamin D treatment in Metabolic Associated Fatty Liver Disease	Vitamin D and Vitamin D receptors are involved in intrahepatic regulation of insulin sensitivity, lipid accumulation, immune response to inflammation, and gut and adipose tissue homeostasis, contributing to NAFLD and NASH pathogenesis.
Coelho et al. [4]	2020	Cross-sectional Study	To investigate the association between serum and dietary antioxidant micronutrients with advanced fibrosis in patients with NAFLD	Hepatic fibrosis is associated with a reduction in serum retinol. A high proportion of NAFLD patients showed a deficiency of retinol, Vitamin C, and selenium, in addition to significant inadequacy of Vitamin A and Vitamin E intake.
Zeng et al. [25]	2020	Animal Intervention Study	To determine the effects of low, medium, and high doses of Vitamin C (15, 30, and 90 mg/kg per day, respectively) when used as prophylaxis and therapy in mice with high-fat-diet-induced NAFLD	Prophylactic administration of a low or medium dose of Vitamin C reduces the risk of NAFLD development. A medium dose of Vitamin C can also ameliorate NAFLD symptoms and improve liver steatosis. However, hepatic injury is pronounced with a high dosage of Vitamin C.
Borges-Canha et al. [7]	2021	Cross-sectional study	To evaluate the association between Vitamin D level of morbidly obese patients and their hepatic function parameters and scores such as FLI and BARD, which are predictors of hepatic steatosis and hepatic fibrosis, respectively	In morbidly obese patients, Vitamin D deficiency is linked with a higher risk of steatohepatitis.
Podszun et al. [6]	2021	Traditional Review	To discuss the influence of Vitamin E (mainly alpha-tocopherol) on redox biomarkers in the context of NAFLD	Reactive species-mediated damage to lipids occurs in NAFLD and NASH, and daily supplementation of alpha-tocopherol is beneficial in alleviating oxidative stress in the liver in NAFLD patients.
Raza et al. [21]	2021	Traditional Review	To provide a broader discussion regarding the role of some vitamins in NAFLD pathophysiology and management	Vitamin deficiency has been correlated with NAFLD severity. Vitamins have anti-inflammatory and insulin-sensitizing benefits in the hepatocytes. Vitamins A, B9, B12, C, D, and E are potential therapeutic options for NAFLD and NASH.

Limitations

Studies included in this review are limited to the English language and a specified time frame. However, some studies, including systematic review and clinical trials conducted before 2016 and those written in other languages, may also be worth reviewing to understand NAFLD pathogenesis and management. In addition, included articles have some notable findings observed in animals but are not widely studied in a more significant number of human subjects. There is also a need for standardized diagnostic criteria and longer follow-up. Information on other vitamins is likewise scarce. Moreover, data supporting the use of vitamin supplements in NAFLD and details regarding their therapeutic and toxic dosage are inadequate. Thus, because of limited evidence and conflicting literature, this remains a subject for future intensive studies.

## Conclusions

Non-Alcoholic Fatty Liver Disease (NAFLD) is characterized as a spectrum of liver conditions with complex pathogenesis. Significant factors leading to this disease include insulin resistance, innate immunity, oxidative stress, genetics, epigenetics, and gut microbiota. In addition, vitamin deficiency has been associated with NAFLD development and severity. Derangement in vitamins is linked to the lipotoxic hepatic environment, altered immune system, oxidative biomolecular damage, dysregulated redox process, unwarranted inflammation, gene mutations, epigenetic modification, and intestinal microbiome changes in NAFLD. While lifestyle modification involving weight loss, increased exercise, and reduced high-calorie diet remains the standard of care for NAFLD, some vitamins also have hepatoprotective benefits. Moreover, Vitamins A, B3, B6, B9, B12, C, D, and E can serve as potential targets for NAFLD therapy, although some are related to adverse effects. On the other hand, current studies regarding their therapeutic and toxic dosages are still insufficient. Also, the associations of other Vitamin B compounds and Vitamin K with NAFLD are still subjects for future research. Because current evidence is still scarce and conflicting, well-designed studies especially involving a larger human population size, standardized diagnostic criteria, uniform drug dose, and longer follow-up, are still necessary to establish the role of vitamins in the pathogenesis and management of NAFLD.
